# Contributing factors in children who present with calcaneal apophysitis

**DOI:** 10.1186/1757-1146-8-S2-O21

**Published:** 2015-09-22

**Authors:** James Alicia, Williams Cylie, Haines Terry

**Affiliations:** 1 Podiatry Department, Peninsula Health Service, Frankston, Vic, 3199, Australia; 2 Physiotherapy Department, Monash University, Frankston, Vic, 3199, Australia; 3 Allied Health Research Unit, Monash Health, Cheltenham, Vic, 3192, Australia

## Background

There are hypothesis that taller children those who are overweight and those restricted ankle range of motion are at risk of developing calcaneal apophysitis. The purpose of this study was to examine associations between pain experienced as a result of calcaneal apophysitis, anthropometric data and foot posture.

## Methods

This study was a cross-sectional study with comparison to normative data. 124 children (males = 72, mean age 10.88 + 1.48 years) with a clinical diagnosis of calcaneal apophysitis were recruited. Normative data was used from Australian and International publications. The baseline measures recorded were: height; weight; waist circumference; BMI; foot posture; and ankle joint range of motion. Univariate and multivariable regression analyses were undertaken to identify factors associated with the severity of pain experienced.

## Results

The children within this study had a higher mean BMI (p<0.001), increased weight (p<0.001), were taller (p<0.001) and had a flatter foot posture (insert stat) compared to normative values. However children demonstrated significantly greater ankle joint range of motion (insert stat) compared to normative values. Multivariable regression analyses identified that older participants (p=0.046) and those who had experienced pain for longer (p=0.043) reported higher pain severity.

## Conclusion

The results of this study indicate that clinicians should reconsider giving a stretching program or heel raises to children presenting with calcaneal apophysitis. An absence in ankle range of motion restriction indicates there is no imbalance in the length of the gastroc soleus complex and the tibia, which is a commonly hypothesized cause of calcaneal apophysitis. The factors that were identified are supportive of theories related to the amount of impact force at the apophysitis, and posture of the foot. These findings indicate that the use of shock absorbing footwear/materials or foot posture interventions may be warranted. Randomised controlled trials are required to test these conclusions.

